# Ethnic and Sociocultural Differences in Ovarian Reserve: Age-Specific Anti-Müllerian Hormone Values and Antral Follicle Count for Women of the Arabian Peninsula

**DOI:** 10.3389/fendo.2021.735116

**Published:** 2021-10-21

**Authors:** Laura Melado, Raquel Vitorino, Carol Coughlan, Leyla Depret Bixio, Ana Arnanz, Ibrahim Elkhatib, Neelke De Munck, Human M. Fatemi, Barbara Lawrenz

**Affiliations:** ^1^ Medical Department, ART Fertility Clinics, Abu Dhabi, United Arab Emirates; ^2^ Medical Department, Advanced Reproductive Technologies (ART) Fertility Clinics, Dubai, United Arab Emirates; ^3^ Biostatistics Department, Consofi Pharma, Dubai, United Arab Emirates; ^4^ Departamento de Biomedicina y Biotecnología, Universidad de Alcalá, Madrid, Spain; ^5^ Medical Department, Women’s University Hospital Tuebingen, Tuebingen, Germany

**Keywords:** Anti-Müllerian hormone, antral follicle count, Arab Peninsula, ovarian reserve, ethnic

## Abstract

**Background:**

Anti-Müllerian hormone (AMH) and antral follicle count (AFC) age-specific reference values form the basis of infertility treatments, yet they were based upon studies performed primarily on Caucasian populations. However, they may vary across different age-matched ethnic populations. This study aimed to describe age-specific serum AMH and AFC for women native to the Arabian Peninsula.

**Methods:**

A retrospective large-scale study was performed including 2,495 women, aged 19 to 50 years, native to the Arabian Peninsula. AMH and AFC were measured as part of their fertility assessment at tertiary-care fertility centres. Age-specific values and nomograms were calculated.

**Results:**

2,495 women were evaluated. Mean, standard deviation and median values were calculated for AMH and AFC by 1-year and 5-years intervals. Median age was 34.81 years, median AMH was 1.76ng/ml and median AFC was 11. From the total group, 40.60% presented with AMH levels below 1.3ng/mL. For women <45 years old, the decrease in AFC was between -0.6/-0.8 per year. Up to 36 years old, the decrease of AMH was 0.1ng/ml. However, from 36 to 40 years old, an accelerated decline of 0.23ng/ml yearly was noted. In keeping with local customs, 71.23% of women wore the hijab and 25.76% the niqab. AMH and AFC were significantly lower for niqab group compared with hijab group (*p*=0.02 and *p*=0.04, respectively).

**Conclusion:**

This is to-date the largest data set on age-specific AMH and AFC values in women from the Arabian Peninsula aiming to increase clinical awareness of the ovarian reserve in this population.

## Introduction

In recent years, Anti-Müllerian hormone (AMH) and antral follicle count (AFC) have become widely used markers for baseline assessment of ovarian reserve, especially preceding assisted reproductive techniques (ART) (1). AMH is used to individualize the treatment regime, not only due to its ability to predict the ovarian response to gonadotropins and the risk of cycle cancellation (2–4), but also as an indicator for oocyte and embryo quality (5, 6), euploidy rate (5, 7) and miscarriage rate (8) and oocyte survival rates following vitrification (9).

Ovarian reserve declines over the reproductive lifespan and has a relevant impact on present and future fertility. This is why AMH and AFC have an important influence on treatment decisions. However, these markers can be influenced by different factors. The so far biggest database on age-specific AMH values, which includes more than 17,000 patients, was published by Seifer et al. in 2011 (10). They report age-specific mean and median AMH values in serum samples from patients, aged 24 to 50 years, obtained from U.S. fertility centers in 37 different states and measured by the first-generation immunoassay DSL. AMH ranges over the life span of a women were published by Kelsey et al. (11) by combining data from 20 studies (3260 data points). Likewise, most studies have evaluated the ovarian reserve parameters in Caucasian populations and only few publications have considered other ethnicities (12–14). However, it has been shown that AMH and AFC varies across different age-matched ethnic populations (14–16). Studies comparing patients from Middle East/North Africa (MENA) region with Caucasian patients described a reduced ovarian reserve and lower number of retrieved oocytes for the MENA population (17, 18). These findings point to significant race-dependent differences in the ovarian reserve and in ovarian ageing (19, 20).

Due to the lack of large-scale age-specific ovarian reserve parameters for women from the Arabian Peninsula and with the aim to close this gap in knowledge, this study describes age-specific AMH and AFC values from women native to the Arabian Peninsula region and evaluates them in the context of the existing sociocultural and religious habits from the Middle East. These values can be used as reference by infertility centers treating patients of similar ethnicity, racial and sociocultural characteristics.

## Methods

### Study Design and Participants

This is a large-scale retrospective, observational study for which data from the clinical documentation program had been extracted and analyzed after the ethical approval had been obtained. Included were data from women, native to the Arabian Peninsula, who had their serum AMH and AFC (sum of small antral follicles in both ovaries) measured as part of their fertility assessment at ART Fertility Clinics Abu Dhabi (UAE), Dubai (UAE) and Muscat (Oman), from May 2015 to November 2019. The Arabian peninsula is composed of the countries Yemen, Oman, Qatar, Bahrain, Kuwait, Saudi Arabia and the United Arab Emirates (21).

Serum AMH concentrations were measured by Elecsys^®^ AMH automated assay (for Cobas 601 platform, Roche^®^) for all patients. Imprecision expected from the assay was <5%, as described by the manufacturer; intra-assay and inter-assay co-efficient of variation for Elecsys^®^ AMH automated assay has been reported as 0.5 – 1.4% and 0.7 – 1.9%, respectively (22). To assess AFC, women underwent transvaginal 2D-sonography (Voluson E8, GE Healthcare, United States) during the menstrual cycle (23). The patients were asked to empty their bladders and were placed in the lithotomy position. The ultrasound-scans were performed by reproductive medicine specialists and a systematic ultrasound technique for AFC measurement was used in order to avoid any bias through different techniques: identification of the ovaries and evaluation of the dimensions in two planes; measurement of the largest follicle in two dimensions; the probe was then positioned along the long axis of the ovary and count the follicles from outer ovarian margin to the opposite margin for both ovaries. The number of follicles in each ovary was combined to obtain the AFC. The number of antral follicles of 2 to 10mm in diameter were counted (24).

A strict dress code is imposed on the women from the Arabian Peninsula due to sociocultural and religious habits in the Middle East region. “Hijab” describes a type of modest clothing with a veil covering the head, neck and chest and “Niqab” is a veil that covers the face while leaving the eyes uncovered (25).

Each patient’s data was recorded only once in the study. Patients, who had been on any hormonal treatment within three months before AMH and AFC measurement, were excluded. Ethical approval was obtained from the Research Ethics Committee (REFA040) of ART Fertility clinic, Abu Dhabi, UAE.

### Statistical Analysis

Patient characteristics are described using mean ± SD, median, minimum, and maximum values for continuous variables, frequencies and percentages for categorical variables. The normality of data distribution was tested through Kolmogorov-Smirnov normality test. Mean ± SD and median values were calculated for AMH and AFC for women ages 19–50 at 1-year intervals (31 age groups in total) and by five age categories (≤25, 26-30, 31-35, 36-40, 41-45, 46-50). Five empirical centiles, including the 5th, 25th, 50th, 75th and 95th were calculated, and nomogram tables were constructed. Graphics based on the percentile age for each variable were drawn using Proc Quantreg. AMH was categorized in 3 groups to evaluate the frequency of low (<1.3 ng/ml), normal (1.3 - 6.24 ng/ml) or high (≥6.25 ng/ml) levels of AMH. The low AMH cut-off level was set at <1.3 ng/mL following the Bologna Criteria (26, 27). The high AMH cut-off level was set at ≥ 6.25 ng/mL, as proposed by Calzada et al. (28) in order to define a cut-off level for PCOS (polycystic ovarian syndrome) patients. Association between dress code and AMH and age were tested using General Linear Model procedure. R-squared were calculated using regression model to measure the strength of the relationship between AMH, AFC, age and BMI. A multiple regression analysis for both AMH and AFC as dependent variables was performed, including the variables studied (age, BMI and dress code) as predictor variables.

As the lower limit of detection for Elecsys assay was 0.01ng/mL, values lower than the detection limit were considered equal to zero. All analyses and the graph plotting were performed using SAS studio.

## Results

**Figure 1:**
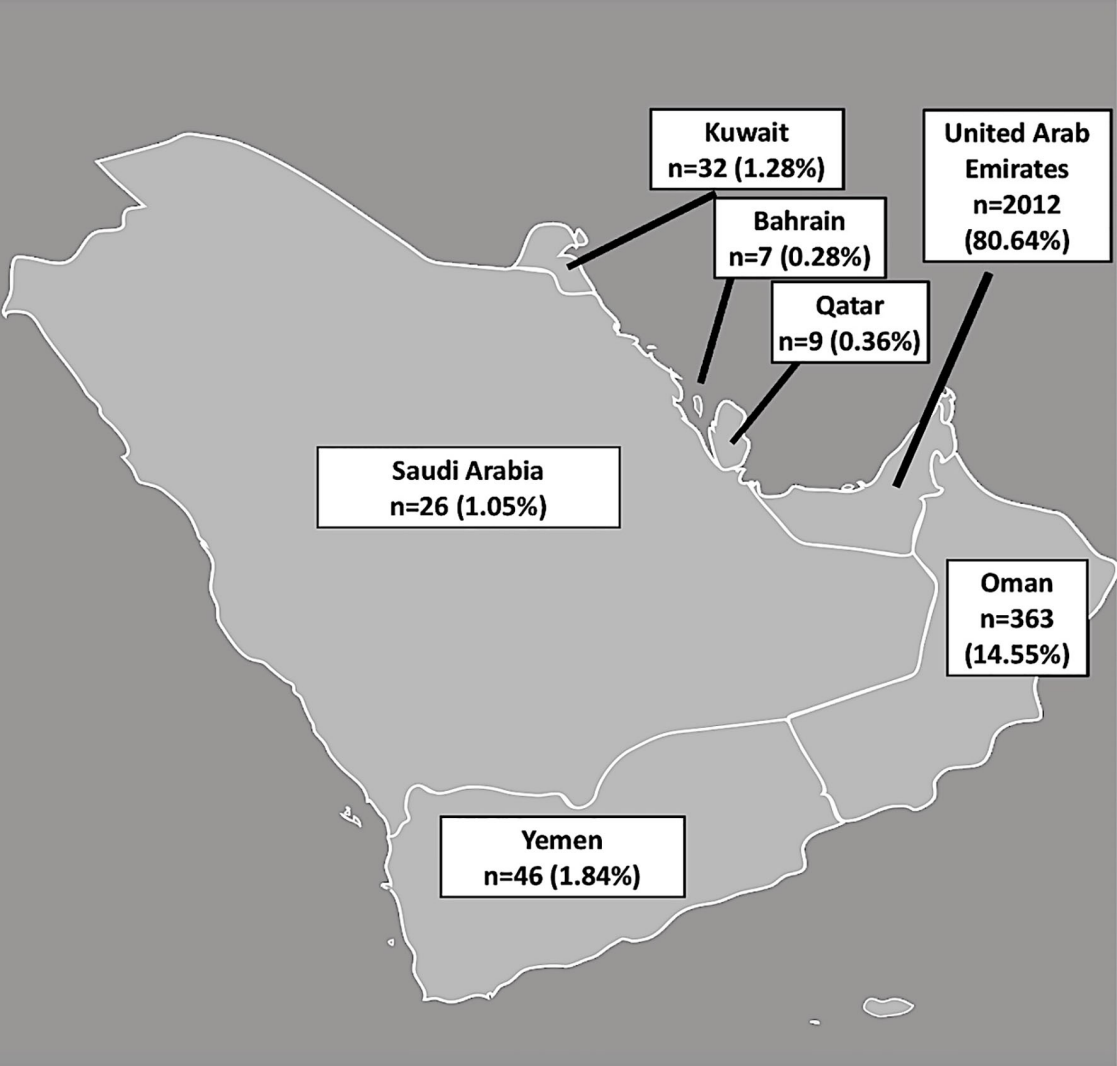
Number and percentage of women included from each country from the Arabian Peninsula.

A total of 2,495 patients, native to the Arabian Peninsula with data on AMH, dress code and BMI were included for analysis. AFC values were available for 2,441 patients. The number and percentage of women included from each country is shown in **Figure 1**.

**Figure 2:**
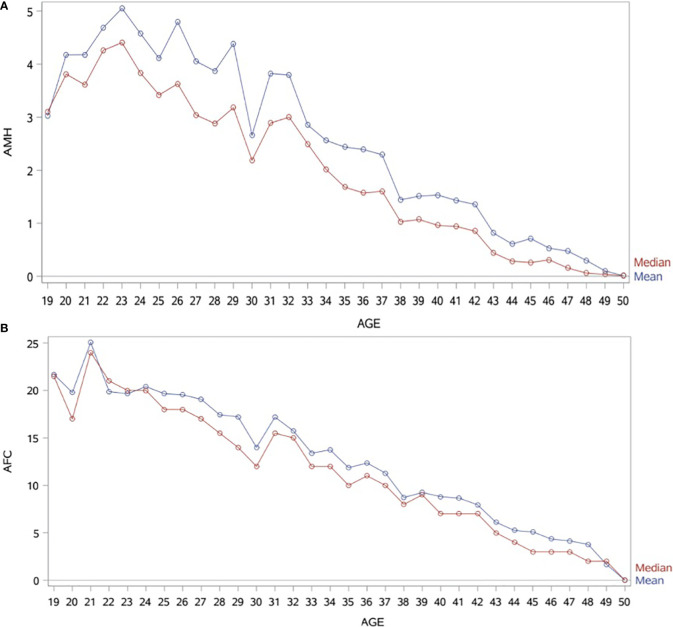
**(A)** Graphical comparisons of AMH mean and median in 1-year intervals. AMH (ng/mL). **(B)** Graphical comparisons of AFC mean and median in 1-year intervals.

**Figure 3:**
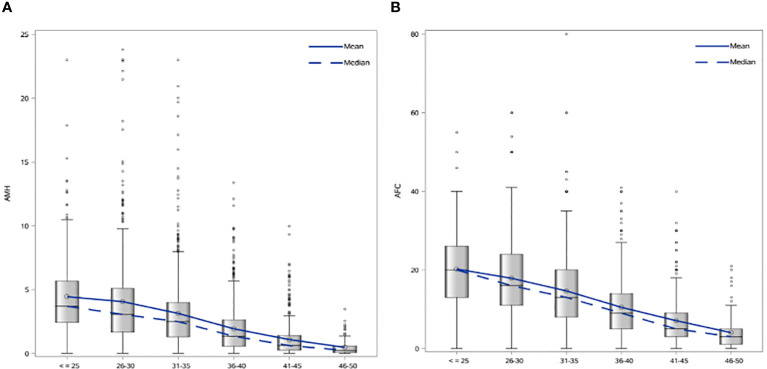
**(A)** Age-specific AMH mean and median values in 5-year intervals. **(B)** Age-specific AFC mean and median values in 5-year intervals.

Patient characteristics were as follows (in mean ± SD): age 34.62 ± 6.61 years, BMI 28.57 ± 5.49 kg/m^2^. The smoking rate among the patients was 2.40%. Most frequent dress code was hijab (71.23%), followed by niqab (25.76%). Mean AMH was 2.59 ± 2.9 ng/mL, whereas median AMH was 1.76 ng/mL. The percentage of women with previous adnexal surgery in the total group analyzed was 11.43%, with a similar distribution between the hijab and niqab groups (12.31% and 14.47%, respectively; *p*=0.704). Patient’ characteristics are displayed in **Table 1**. Single-year specific median, mean and SD for AMH and AFC are summarized in **Table 2** and graphical comparisons of AMH and AFC in 1-year intervals are shown in **Figures 2A, B** respectively; **Table 3** and **Figures 3A, B** include median, mean, SD values, and average decrease per age category. The decrease of AMH was highest in the age group 36-40 (AMH median: -1.15 ng/mL between age 36 and 40), showing a yearly decrease of -0.23 ng/mL.

**Table 1:** Demographic characteristics.

	n	Percentage	Mean	SD	Median	Min - Max
**AGE (years)**	2495		34.62	6.61	34.81	18.68-49.60
**INFERTILITY DURATION (years)**	2489		3.865	3.71	3.00	0-29
**BMI (kg/m^2^)**	2495		28.57	5.49	28.03	14.34-53.42
**AMH (ng/mL)**	2495		2.59	2.90	1.76	0.01-23.80
**AFC**	2441		12.72	9.43	11.00	0-80
**DRESS CODE**	2395					
Hijab	1706	71.23%				
Nikab	617	25.76%				
None	72	3.01%				
**SMOKING**	2461					
No	2399	97.48%				
Yes	59	2.40%				
Ex-smoker	3	0.12%				
**TYPE OF INFERTILITY**	2494					
Primary	1045	41.88%				
Secondary	1392	55.79%				
Oncologic fertility preservation	3	0.12%				
Gestational desire because of genetic disease	48	1.92%				
No oncological fertility preservation	6	0.04%				
**PREVIOUS ADNEXAL SURGERY**	2495					
No	2210	88.57%				
Yes	285	11.43%				

**Table 2:** Single-age specific AMH mean with standard deviation (SD) and median values obtained by Elecsys AMH immunoassay, and single-age specific AFC mean with standard deviation (SD) and median.

Age	n AMH	AMH Mean	SD	AMH Median	n AFC	AFC Mean	SD	AFC Median
19	10	3.03	1.25	3.10	10	21.7	6	21.5
20	10	4.17	2.14	3.81	9	19.8	9	17
21	17	4.18	2.31	3.61	16	25.1	10	24
22	33	4.69	3.09	4.26	33	19.9	10	21
23	40	5.05	3.66	4.41	40	19.7	8	20
24	54	4.57	3.27	3.83	53	20.4	10	20
25	90	4.11	3.18	3.42	88	19.6	10	18
26	74	4.80	4.20	3.63	72	19.5	9	18
27	96	4.05	3.66	3.04	93	19.1	11	17
28	89	3.87	3.71	2.88	88	17.4	9	15.5
29	109	4.38	4.26	3.18	108	17.2	11	14
30	100	2.66	1.97	2.19	98	14.0	9	12
31	133	3.82	3.80	2.89	128	17.2	12	15.5
32	112	3.79	3.37	3.00	111	15.7	10	15
33	112	2.85	2.29	2.50	111	13.4	8	12
34	126	2.56	2.33	2.01	125	13.7	7	12
35	124	2.44	2.51	1.68	124	11.9	8	10
36	113	2.39	2.35	1.57	108	12.4	8	11
37	132	2.29	2.23	1.61	130	11.3	7	10
38	115	1.45	1.52	1.03	114	8.7	6	8
39	122	1.51	1.40	1.07	119	9.2	6	9
40	123	1.53	1.54	0.96	118	8.8	7	7
41	107	1.43	1.53	0.94	101	8.7	7	7
42	122	1.36	1.56	0.85	121	7.9	6	7
43	107	0.82	0.92	0.44	104	6.1	5	5
44	78	0.61	0.84	0.29	77	5.3	5	4
45	56	0.71	1.46	0.26	53	5.1	6	3
46	46	0.53	0.54	0.31	44	4.3	4	3
47	20	0.47	0.61	0.16	20	4.2	4	3
48	18	0.30	0.56	0.06	18	3.8	5	2
49	6	0.10	0.12	0.04	6	1.7	1	2
50	1	0.01	.	0.01	1	0	.	0

Units, ng/mL; n, number of patients; age, years.

**Table 3:** Median, mean, SD values, and average decrease per age category for serum AMH levels (ng/mL) and AFC.

Age Category (years)	n	AMH Mean	AMH Median	AMH Median Decrease Per Age Category	Yearly AMH median Decrease	AFC Mean	AFC Median	AFC Median Decrease Per Age Category	Yearly AFC median Decrease
**≤ 25**	205	4.45	3.70			20.22	20		
**26-30**	469	4.06	3.06	- 0.64	-0.13	17.88	16	-4	-0.8
**31-35**	596	3.14	2.50	- 0.57	-0.11	14.67	13	-3	-0.6
**36-40**	611	1.94	1.35	- 1.15	-0.23	10.49	9	-4	-0.8
**41-45**	499	1.09	0.62	- 0.73	-0.15	7.09	5	-4	-0.8
**46-50**	115	0.48	0.24	- 0.38	-0.08	4.04	3	-2	-0.4

Bold values are the age categories.

AMH and AFC values of 5^th^, 25^th^, 50^th^, 75^th^ and 95^th^ centiles are shown as **Supplementary Data** (**Supplementary Tables 1**, **2** and **Supplementary Figures 1**, **2**). Correlation (R-square) for AMH and age was 18.8% and 25.9% for AFC and age (**Supplementary Figure 3**). AMH and AFC showed a R-square of 57.6%. Very low R-square was found for BMI and AMH (R-square=0.08%).

**Figure 4:**
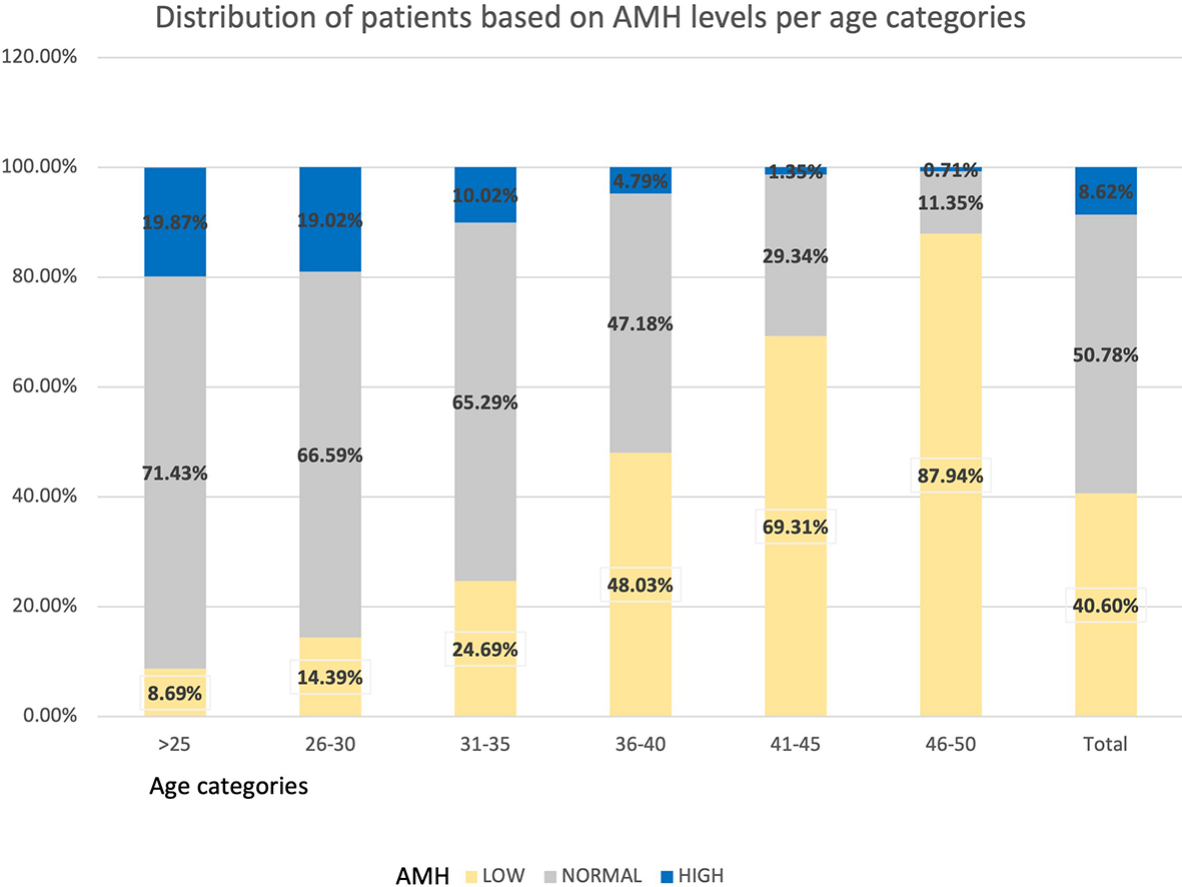
Distribution of patients based on AMH levels per age categories.

The distribution of women based on their levels of AMH is shown in **Table 4** and **Figure 4**. In the total population, 40.60% of women presented AMH serum levels <1.3 ng/mL, 50.78% between 1.3 - 6.24 ng/mL, and 8.62% had AMH ≥6.25 ng/mL.

**Table 4:** Distribution of patients based in their levels of AMH (ng/mL).

AGE	Low (AMH<1.3 ng/mL)	Normal (AMH 1.3-6.24 ng/mL)	High (AMH≥6.25 ng/mL)	Total
**≤ 25**	8.69%	71.43%	19.87%	6.45%
**26-30**	14.39%	66.59%	19.02%	17.27%
**31-35**	24.69%	65.29%	10.02%	22.40%
**36-40**	48.03%	47.18%	4.79%	23.45%
**41-45**	69.31%	29.34%	1.35%	20.76%
**46-50**	87.94%	11.35%	0.71%	5.65%
**Total**	40.60%	50.78%	8.62%	100.00%

Bold values are the age categories.

Data on dress code was available for 2,323 women. There was no difference in age between the two groups of dress code (Hijab versus Niqab), but AMH serum levels and AFC were significantly lower for patients wearing the niqab (*p*=0.043 and *p*=0.021, respectively). The results are shown in **Table 5**.

**Table 5:** Distributions regarding conservative dress code for Arab population.

	n	HIJAB (mean ± SD)	NIQAB (mean ± SD)	*p* value
**AGE (years)**	2323*	34.59 ± 6.55	34.84 ± 6.97	ns
**AFC**	2273	13.01 ± 9.58	11.97 ± 9.78	0.021
**AMH (ng/mL)**	2323	2.63 ± 2.84	2.45 ± 2.93	0.043

*Patients with dress code and AMH data included for the analysis, out of the total population (n=2495). n, number of patients included; SD, standard deviation; ns, non significative.

A multiple regression analysis for both AMH and AFC as dependent variables was performed, including age, BMI and dress code as predictor variables. Age and BMI showed a significant correlation to AFC (*p*< 0.0001 for both predictor variables) and to AMH (*p*<0.0001 and *p*=0.004, respectively). Regarding the dress code, a significant negative correlation was observed for the niqab dress code to AFC (*p*=0.025), however, not significant for AMH (*p*=0.22) (**Table 6**).

**Table 6:** Multiple regression analysis for AFC and AMH as dependent variables, including age, BMI and dress code as predictor variables.

AFC (n=2273)	Coefficient	Std. Err.	95% CI	P value
**Age**	-0.758	0.026	-0.808/-0.707	<0.0001
**BMI**	0.187	0.031	0.126/-0.249	<0.0001
**Dress Code (Hijab – Niqab)**	-0.860	0.382	-1.609/-0.110	0.025
**AMH (n=2323)**	**Coefficient**	**Std. Err.**	**95% CI**	**P value**
**Age**	-0.194	0.008	-0.210/-0.178	<0.0001
**BMI**	0.028	0.010	0.009/-0.048	0.004
**Dress Code (Hijab – Niqab)**	-0.147	0.121	-0.384/-0.089	0.223

Std.Err., Standard Error; 95% CI, 95% Confidence Interval; BMI, Body Mass Index.

## Discussion

Whereas it is well known that age is the most significant factor to impact the ovarian reserve, the influence of other factors is often less considered and/or neglected, especially in populations less included in research. To the best of our knowledge, this is the first large-scale retrospective data set reporting age, BMI and dress-code in relation to AMH and AFC values specific to women native to the Arabian Peninsula, with all AMH measurements performed with 3^rd^-generation automated assay (Elecsys, Roche^®^) in a single laboratory. In this specific study population, the peak AMH level (median AMH of 4.41 ng/ml) was exhibited at an age of 23 years, a finding which is in line with the data from Kelsey et al. (11), who also demonstrated an AMH peak at the age of 24.5 years and described lower AMH levels in the population below 24 years of age. A large percentage of women (40.60%) from the herein presented study population were affected by a low ovarian reserve, expressed as AMH serum levels <1.3ng/mL (26). While an age-dependent physiological decrease for both ovarian reserve markers was expected at any given age, it is worth noting that AMH presented an accelerate decrease for the age category of 36-40 years old. This significant decrease over those five years is in keeping with the already well recognized decrease in ovarian reserve and fertility for women above 35 years old (29). The multiple regression analysis showed that age and BMI were strong predictor variables for the ovarian reserve markers. The dress code was a significant predictor variable for AFC, however, no significant value was reached for AMH.

There is growing evidence that ovarian reserve is, besides age, influenced by several factors including the sociocultural environment, habits and ethnicity. Previous publications have demonstrated, that Asian, African American, Hispanic, Indian American have lower live birth rates (LBR) and higher miscarriage rates after ART when compared to a reference population of white/Caucasian women (30–37). Despite the fact that the Arabian culture is a pro-family society and families commonly have a higher number of children compared to couples in Western societies (38, 39), there is a high prevalence of infertility in the MENA region (40) and very limited knowledge about ovarian reserve parameters and ART outcomes in the Arab population.

Women native to the MENA (Middle East North Africa) region, which is a region comprising 17 countries with multiple ethnicities (The Middle East Population 2021 (Demographics, Maps, Graphs), no date), are only included into few studies (17, 18, 35, 37). Feichtinger et al. (17) found a significantly lower oocyte yield in MENA patients compared to their European counterparts, despite being of younger age and having a higher prevalence of PCOS. Salem et al. (37) described poorer IVF outcomes with decreased live birth rates per blastocyst transfer and increased miscarriage rates in MENA women compared to Caucasian women. In the study of Jayaprakasan et al. (35), the Middle Eastern women presented the lowest LBR (21.4%), yet only 14 women were included for the analysis. Similar data are described by Tabbalat et al. (18), who compared the IVF outcomes for infertile couples from the Arabian Peninsula to Caucasian couples from the U.S. The Arabian Peninsula women showed statistically higher FSH levels, lower AMH levels and lower AFC, as well as lower number of mature oocytes. These results suggest that Arabian Peninsula ethnicity is associated with lower ovarian reserve and ovarian response, and these findings are corroborated by the AMH and AFC values from the herein presented data when compared to the ovarian reserve parameters described in other ethnicities. Loy et al. (12) presented percentiles and nomograms for AMH and AFC for 1,009 Chinese sub-fertile women, using Gen II ELISA assay for AMH. Considering the possible variations due to the different assays used [serum AMH concentrations seem to be 20% lower when Elecsys is compared with conventional AMH Gen II assays (41)], AMH values for Chinese ethnicity seem clearly higher than for the Arab population. Compared to Japanese population, the median AMH values presented in this study are 38.4% lower using Access assay (14). This percentage is far from the 10% expected due to the different assays used (42). Likewise, the percentage of women with low AMH values (<1.3 ng/mL) in the herein presented study is 40.60%, a high rate when compared to 13.2% described for a group of 423 infertile women from Pakistan (13) and apparently, these disparities are more distinct in the younger groups (43).

The reduced ovarian reserve identified in the Middle Eastern ethnical group highlights the influence of ethnicity, sociocultural, religious, environmental and genetic factors on the ovarian reserve and consequently on fertility (20). The high rates of consanguineous marriages, which amount up to 80% in certain regions in the Middle East, have been related to a reduced ovarian reserve (20, 44). Environmental factors have an impact as well, e.g. vitamin D deficiency, which has one of the world’s highest prevalence rates in the Middle East (45). Lately, the positive impact of 1α,25-dihydroxyvitamin D3, which is the biologically active form, on the ovarian reserve has been demonstrated in the ovarian tissue of rhesus macaques (46, 47) as well as in patients, who received a single, high dosage of vitamin D at the beginning of their cycle (48). It is important to acknowledge that the dress-code of women correlates with the extent of vitamin D deficiency (49, 50) and most women of the Arabian Peninsula are following a concealing dress-code with coverage of large parts of the skin due to sociocultural/religious habits. Arefi et al. (19) previously showed a negative influence of the dress-code on the ovarian reserve parameters. The data of the present study support the previous findings as, indeed, women using the most covering dress code (Niqab) have significantly lower values for AMH and AFC compared to women with a less concealing dress-code. Furthermore, dress code was a significant predictive variable for AFC in the regression analysis. No significant result was observed for the association with AMH, hence, this discrepancy could be explained by the lower number of included AMH results and a non-normal distribution for the AMH values. Body weight and BMI are factors which exhibit a negative impact on the ovarian reserve parameters and it is well known that the prevalence of obesity (BMI>30 Kg/m^2^) in adults aged 18 and above in the Arabian Peninsula are above 40% (51). In line with these data, the women included in the herein study presented a median BMI of 28.03 Kg/m^2^ and the regression analysis showed BMI as a strong predictive variable for both ovarian reserve parameters (AFC: *p*< 0.0001; AMH: *p*=0.004).

Although the retrospective design might be considered as a limitation, the main strengths of the present study are firstly, the large number of included women with similar geographical, sociocultural/religious and ethnical similarities; and secondly, the use of a 3rd-generation automated assay (Elecsys, Roche^®^) for the AMH analysis, performed in a single clinical reference laboratory, leading to an improved accuracy and homogeneity of the results. Further on, this study provides median reference values in addition to mean values for more adequate information regarding AMH and AFC values, as data are non-normally distributed.

This study presents age and dress code specific AMH and AFC in women native to the Arabian Peninsula, facilitating an increased understanding of ovarian reserve in this population and the rates of low, normal and high ovarian reserve. Reproductive specialists should be aware of the influence of ethnicity on the ovarian reserve and to adopt region specific counseling strategies and personalize treatment plans accordingly.

## Data Availability Statement

The original contributions presented in the study are included in the article/**Supplementary Material**. Further inquiries can be directed to the corresponding author.

## Ethics Statement

The study involving human participants were reviewed and approved by the Research Ethics Committee (REFA040) of ART Fertility clinic, Abu Dhabi, UAE. The patients/participants provided their written informed consent to participate in this study.

## Author Contributions

LM, HF, and BL contributed to the study conception and design, data acquisition, analysis, interpretation of data, and drafting of the bulk of the article. LB contributed to the analysis and interpretation of data, conception and design of the statistical analysis. RV, CC, AA, IE, and ND contributed to the data acquisition and updated bibliography. All authors contributed to the article and approved the submitted version.

## Conflict of Interest

The authors declare that the research was conducted in the absence of any commercial or financial relationships that could be construed as a potential conflict of interest.

## Publisher’s Note

All claims expressed in this article are solely those of the authors and do not necessarily represent those of their affiliated organizations, or those of the publisher, the editors and the reviewers. Any product that may be evaluated in this article, or claim that may be made by its manufacturer, is not guaranteed or endorsed by the publisher.

## Supplementary Material

Supplementary Table 1 AMH (ng/mL) by centile and age.

Supplementary Table 2 AFC by centile and age.

Supplementary Figure 1 Centiles of Anti-Mullerian hormone (ng/mL) by age.

Supplementary Figure 2 Centiles of AFC by age.

Supplementary Figure 3 Relations between AMH, AFC and age for the total population (n=2495). Left, relation between AMH values and age (R-square = 0,188). Right, relation between total AFC and age (R-square = 0,259).
