# Supplementary material for: Ethnic and Sociocultural Differences in Ovarian Reserve: Age-Specific Anti-Müllerian Hormone Values and Antral Follicle Count for Women of the Arabian Peninsula
Source: Front Endocrinol (Lausanne). 2021 Oct 21;12:735116. doi: 10.3389/fendo.2021.735116 (PMC8567992; doi:10.3389/fendo.2021.735116)
Supplement: Supplementary Table 1 — AMH (ng/mL) by centile and age. [file Table_1.docx]

SUPPLEMENTARY TABLE 1. AMH (ng/mL) by centile and age.

| Age | n | 5^th^ | 25^th^ | 50^th^ | 75^th^ | 95^th^ |
| --- | --- | --- | --- | --- | --- | --- |
| 19 | 10 | 0.96 | 2.24 | 3.1 | 3.57 | 5.34 |
| 20 | 10 | 2.08 | 3.34 | 3.81 | 4.17 | 9.8 |
| 21 | 17 | 0.94 | 2.96 | 3.61 | 4.92 | 10.65 |
| 22 | 33 | 0.56 | 2.59 | 4.26 | 5.78 | 11.68 |
| 23 | 40 | 0.3 | 2.84 | 4.41 | 6.13 | 12.6 |
| 24 | 54 | 0.89 | 2.28 | 3.83 | 5.66 | 12.79 |
| 25 | 90 | 0.76 | 2 | 3.42 | 5.48 | 9.3 |
| 26 | 74 | 0.5 | 1.81 | 3.63 | 6.33 | 12.69 |
| 27 | 96 | 0.39 | 1.74 | 3.04 | 4.81 | 12.03 |
| 28 | 89 | 0.63 | 1.92 | 2.88 | 4.62 | 10.32 |
| 29 | 109 | 0.22 | 1.56 | 3.18 | 5.68 | 11.92 |
| 30 | 100 | 0.19 | 1.23 | 2.19 | 3.87 | 6.33 |
| 31 | 133 | 0.32 | 1.61 | 2.89 | 4.62 | 9.32 |
| 32 | 112 | 0.09 | 1.87 | 3 | 4.78 | 9.01 |
| 33 | 112 | 0.31 | 1.33 | 2.5 | 3.67 | 7.94 |
| 34 | 126 | 0.27 | 1.05 | 2.01 | 3.32 | 6.18 |
| 35 | 124 | 0.13 | 0.72 | 1.68 | 3.21 | 6.6 |
| 36 | 113 | 0.1 | 0.81 | 1.57 | 3.13 | 8.1 |
| 37 | 132 | 0.15 | 0.69 | 1.61 | 3.07 | 6.72 |
| 38 | 115 | 0.01 | 0.31 | 1.03 | 2.07 | 4.43 |
| 39 | 122 | 0.02 | 0.39 | 1.07 | 2.34 | 4.57 |
| 40 | 123 | 0.08 | 0.41 | 0.96 | 2.16 | 4.65 |
| 41 | 107 | 0.07 | 0.35 | 0.94 | 2.17 | 5.34 |
| 42 | 122 | 0.01 | 0.36 | 0.85 | 1.74 | 4.4 |
| 43 | 107 | 0.02 | 0.23 | 0.44 | 1.14 | 2.62 |
| 44 | 78 | 0.01 | 0.1 | 0.29 | 0.72 | 1.98 |
| 45 | 56 | 0.01 | 0.1 | 0.26 | 0.725 | 2.55 |
| 46 | 46 | 0.01 | 0.15 | 0.31 | 0.72 | 1.55 |
| 47 | 20 | 0.01 | 0.04 | 0.16 | 0.86 | 1.8 |
| 48 | 18 | 0.01 | 0.02 | 0.06 | 0.31 | 2.12 |
| 49 | 6 | 0.01 | 0.01 | 0.04 | 0.24 | 0.28 |
| 50 | 1 | 0.01 | 0.01 | 0.01 | 0.01 | 0.01 |
