# Supplementary material for: Ethnic and Sociocultural Differences in Ovarian Reserve: Age-Specific Anti-Müllerian Hormone Values and Antral Follicle Count for Women of the Arabian Peninsula
Source: Front Endocrinol (Lausanne). 2021 Oct 21;12:735116. doi: 10.3389/fendo.2021.735116 (PMC8567992; doi:10.3389/fendo.2021.735116)
Supplement: Supplementary Table 2 — AFC by centile and age. [file Table_2.docx]

SUPPLEMENTARY TABLE 2: AFC by centile and age.

| Age | n | 5^th^ | 25^th^ | 50^th^ | 75^th^ | 95^th^ |
| --- | --- | --- | --- | --- | --- | --- |
| 19 | 10 | 0.96 | 2.240 | 3.100 | 3.570 | 5.340 |
| 20 | 9 | 2.08 | 3.340 | 3.810 | 4.170 | 9.800 |
| 21 | 16 | 0.94 | 2.960 | 3.610 | 4.920 | 10.650 |
| 22 | 33 | 0.56 | 2.590 | 4.260 | 5.780 | 11.680 |
| 23 | 40 | 0.30 | 2.835 | 4.405 | 6.130 | 12.595 |
| 24 | 53 | 0.89 | 2.280 | 3.830 | 5.660 | 12.790 |
| 25 | 88 | 0.76 | 2.000 | 3.420 | 5.480 | 9.300 |
| 26 | 72 | 0.50 | 1.810 | 3.630 | 6.330 | 12.690 |
| 27 | 93 | 0.39 | 1.740 | 3.040 | 4.810 | 12.030 |
| 28 | 88 | 0.63 | 1.920 | 2.880 | 4.620 | 10.320 |
| 29 | 108 | 0.22 | 1.560 | 3.180 | 5.680 | 11.920 |
| 30 | 98 | 0.19 | 1.230 | 2.190 | 3.870 | 6.325 |
| 31 | 128 | 0.32 | 1.610 | 2.890 | 4.620 | 9.320 |
| 32 | 111 | 0.09 | 1.865 | 3.000 | 4.780 | 9.010 |
| 33 | 111 | 0.31 | 1.325 | 2.495 | 3.670 | 7.940 |
| 34 | 125 | 0.27 | 1.050 | 2.010 | 3.320 | 6.180 |
| 35 | 124 | 0.13 | 0.715 | 1.680 | 3.210 | 6.600 |
| 36 | 108 | 0.10 | 0.810 | 1.570 | 3.130 | 8.100 |
| 37 | 130 | 0.15 | 0.685 | 1.605 | 3.070 | 6.720 |
| 38 | 114 | 0.01 | 0.310 | 1.030 | 2.070 | 4.430 |
| 39 | 119 | 0.02 | 0.390 | 1.070 | 2.340 | 4.570 |
| 40 | 118 | 0.08 | 0.410 | 0.960 | 2.160 | 4.650 |
| 41 | 101 | 0.07 | 0.350 | 0.940 | 2.170 | 5.340 |
| 42 | 121 | 0.01 | 0.360 | 0.850 | 1.740 | 4.400 |
| 43 | 104 | 0.02 | 0.230 | 0.440 | 1.140 | 2.620 |
| 44 | 77 | 0.01 | 0.100 | 0.285 | 0.720 | 1.980 |
| 45 | 53 | 0.01 | 0.095 | 0.260 | 0.725 | 2.550 |
| 46 | 44 | 0.01 | 0.150 | 0.310 | 0.720 | 1.550 |
| 47 | 20 | 0.01 | 0.035 | 0.155 | 0.855 | 1.795 |
| 48 | 18 | 0.01 | 0.020 | 0.060 | 0.310 | 2.120 |
| 49 | 6 | 0.01 | 0.010 | 0.035 | 0.240 | 0.280 |
| 50 | 1 | 0.01 | 0.010 | 0.010 | 0.010 | 0.010 |
