# Supplementary figures and images for: Ethnic and Sociocultural Differences in Ovarian Reserve: Age-Specific Anti-Müllerian Hormone Values and Antral Follicle Count for Women of the Arabian Peninsula
Source: Front Endocrinol (Lausanne). 2021 Oct 21;12:735116. doi: 10.3389/fendo.2021.735116 (PMC8567992; doi:10.3389/fendo.2021.735116)

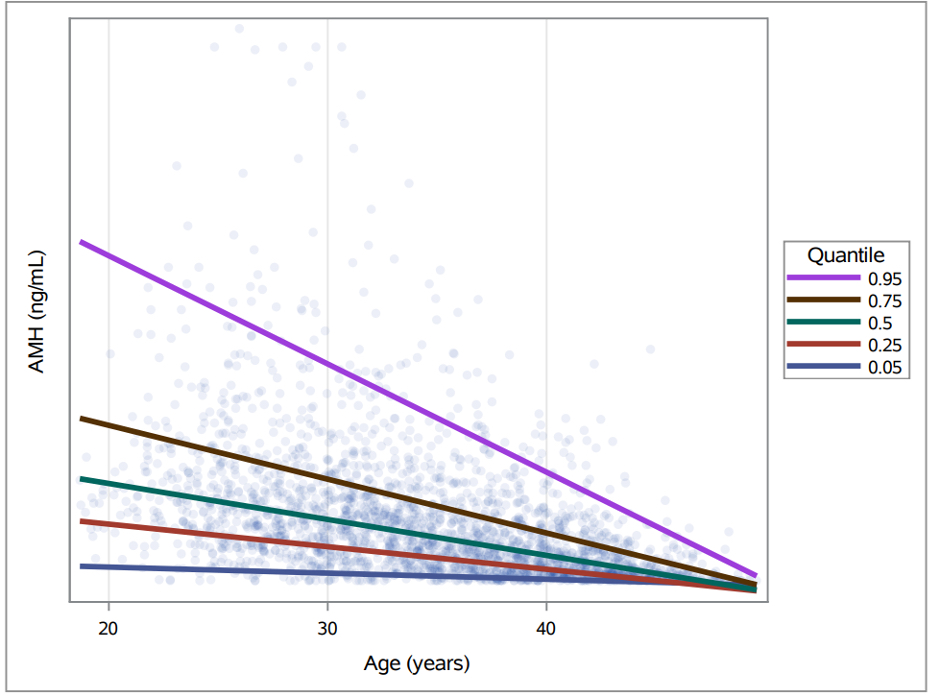

Supplement: Supplementary Figure 1 — Centiles of Anti-Mullerian hormone (ng/mL) by age. [file Image_1.jpg]

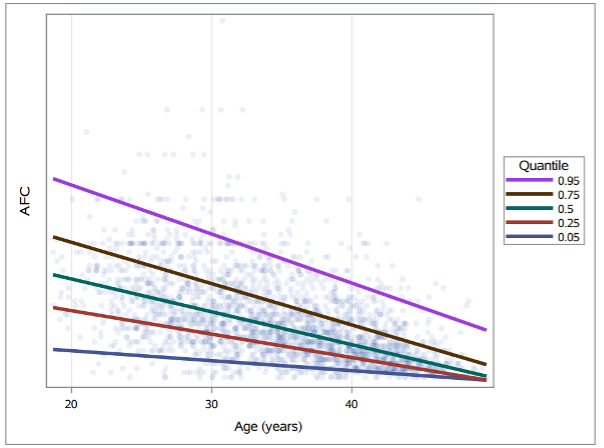

Supplement: Supplementary Figure 2 — Centiles of AFC by age. [file Image_2.jpg]

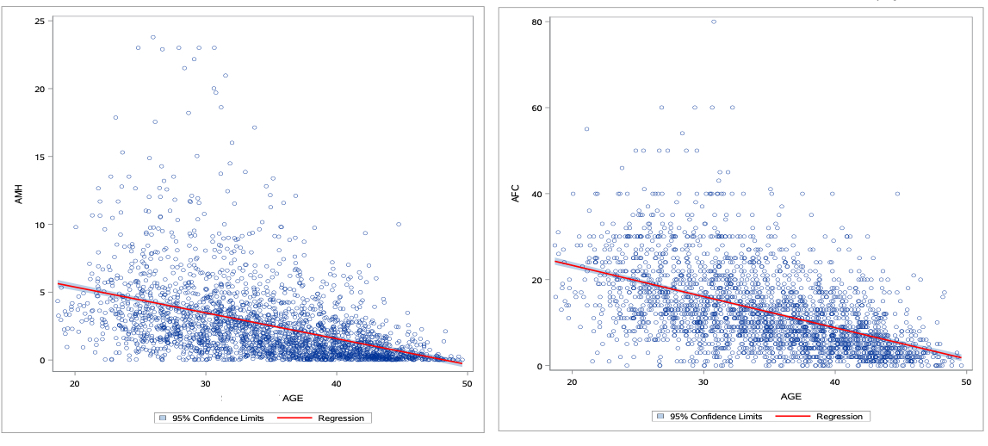

Supplement: Supplementary Figure 3 — Relations between AMH, AFC and age for the total population (n=2495). Left, relation between AMH values and age (R-square = 0,188). Right, relation between total AFC and age (R-square = 0,259). [file Image_3.jpg]
